# Vasculitic Tibial Mononeuropathy Associated with Inherited Immune Dysregulation: A Review of Tibial Mononeuropathies with Electrodiagnostic Considerations

**DOI:** 10.1155/2021/7161757

**Published:** 2021-11-12

**Authors:** James Liu, Yue Ding, Sandra Camelo-Piragua, James Richardson

**Affiliations:** ^1^University of Michigan, Ann Arbor, USA; ^2^University of Toledo, Toledo, USA

## Abstract

Compressive tibial mononeuropathies are uncommon and can be caused by conditions including posterior compartment syndrome, soleal sling syndrome, and tarsal tunnel syndrome. Therefore, it is critical to consider noncompressive etiologies when a tibial mononeuropathy is suspected. This is a patient with a history of rare inherited immune dysregulation that presented to the electrodiagnostic laboratory with severe neuropathic pain in the right foot associated with plantarflexion weakness, concerning for a tibial mononeuropathy. However, the patient's clinical presentation and results on electrodiagnostic testing were not consistent with any of the above entities. Therefore, noncompressive etiologies of tibial mononeuropathies such as vasculitis had to be considered. The patient subsequently underwent sural nerve biopsy which confirmed small-vessel vasculitis as the cause of the tibial mononeuropathy. She was then started on appropriate immunosuppressive treatment which resulted in significant pain relief and was discharged home. This case highlights the importance of considering noncompressive causes of tibial nerve injury. Compressive and vasculitic tibial mononeuropathies along with their electrodiagnostic considerations are reviewed. Furthermore, this case highlights the critical role of the electromyographer and ability to maximize the impact on patient care through a solid foundation in anatomy, pathophysiology, and electrodiagnosis blended with clinical acumen.

## 1. Introduction

Tibial mononeuropathies due to compressive etiologies include posterior compartment syndrome, soleal sling syndrome, and tarsal tunnel syndrome. However, compressive tibial nerve injuries are uncommon since the nerve travels deep in the lower extremity and is well protected by soft tissue [1]. Therefore, when a tibial mononeuropathy is suspected, it is uniquely important to also consider noncompressive etiologies such as vasculitis. The tibial nerve is often affected in small-vessel vasculitis-related neuropathy and is second in frequency only to the peroneal nerve [2]. Due to differences in pathophysiology, each of the above causes of tibial mononeuropathy has distinct electrodiagnostic findings. This case report highlights a patient who was found to have a vasculitic tibial mononeuropathy through clinical evaluation and electrodiagnostic testing which subsequently led to appropriate medical treatment and favorable patient outcome.

## 2. Case Presentation

A 29-year-old female presented with a 1-year history of progressively worsening right foot pain described as “crushing and burning” radiating into the posterior calf but sparing the heel. She had a significant history of actin-related protein complex subunit 1B gene mutations, critical for actin cytoskeleton remodeling, causing immune dysregulation with recurrent infections and ulcers. Her prescribed neuropathic pain medications and opioids had not provided adequate relief. She also reported numbness over the plantar aspect of the foot, again sparing the heel. A couple weeks prior to presentation, she began noticing ankle plantarflexion and toe flexion weakness on the right. The patient denied trauma to the right lower limb or radiating lumbar pain.

On examination, there was no gross muscle atrophy. There were multiple well-healed skin ulcerations over the anterior tibial regions. The patient had full strength in the left lower limb. On the right, she had difficulty with toe flexion. Furthermore, ankle plantarflexion was easily overcome on manual muscle testing when the patient was lying supine, indicating marked weakness. Sensation to light touch on the right was diminished over the plantar surface of the foot sparing the heel. The patient perceived a maximally struck 128 Hz tuning fork for >10 seconds at the first metatarsal-phalangeal joints bilaterally. Reflexes were 2+ at the patellae and left Achilles but absent at the right Achilles.

The patient underwent MRI of the right foot without contrast which showed nonspecific muscular edema suggestive of nonspecific myositis or acute denervation, but no other abnormalities. Initially, she was evaluated by Neurology and Pain Medicine services who found her symptoms to be consistent with complex regional pain syndrome. She was then evaluated by Rheumatology who recommended an electrodiagnostic evaluation concerning vasculitis.

Nerve conduction studies and needle electromyography were performed (Table 1). The sural nerves showed borderline normal amplitudes with normal latencies. The right superficial peroneal sensory amplitude was low normal and decreased on the left. The mixed nerve medial plantar responses were absent bilaterally. The peroneal motor amplitudes were low normal bilaterally. The tibial motor amplitude was mildly diminished on the left and markedly decreased on the right. Needle electromyography demonstrated abnormal spontaneous activity which was most marked in the distal tibial musculature. There was no evidence of axonal continuity to the intrinsic musculature of the forefoot.

Taken together, there was electrodiagnostic evidence of a generalized length-dependent sensorimotor polyneuropathy which was predominantly axonal in nature. There was also a superimposed tibial mononeuropathy on the right, likely localizing just proximal to the tibial take-off branches to the soleus muscle. The findings were consistent with the patient's report of recent severe pain involving the right foot accompanied by ankle plantarflexion and toe flexion weakness.

**Figure 1:**
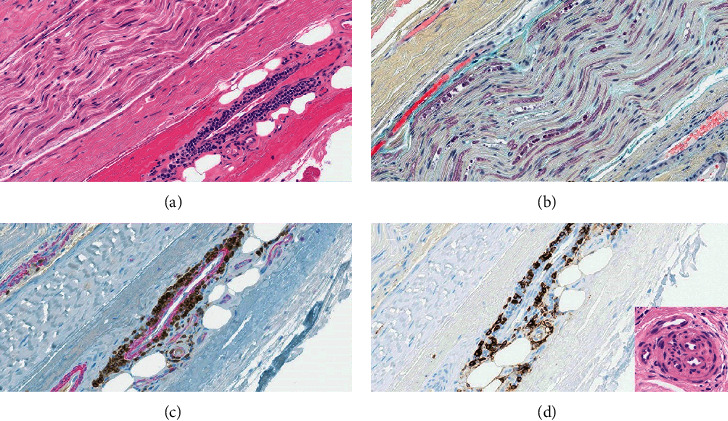
Right sural nerve biopsy. (a) Hematoxylin and eosin staining shows a small caliber vessel in the perineurium surrounded and focally invaded by mononuclear cells, indicative of active ongoing vasculitis. On the top left corner, the longitudinal section of the nerve with significant paucity of myelinated axons. (b) MOVAT pentachrome staining shows several degenerating axons (purple globules), indicative of active ongoing axonopathy; in addition, there is a significant loss of myelinated axons with secondary endoneurial fibrosis (pale-tan material around remaining myelinated axons). (c) Double immunostaining CD3 (brown) smooth muscle actin (red) shows several T cells surrounding, focally invading, and disrupting the vessel wall. (d) CD20 shows admixed B cells. Inset shows the cross section of a reanalyzed vessel indicative of remote, healed vascular injury.

The patient was subsequently referred for a right sural nerve biopsy (Figure 1). Biopsy confirmed the presence of chronic and ongoing small-vessel vasculitis with associated axonopathy. She underwent treatment with three days of pulse steroids followed by additional high-dose steroids and IVIG. Near discharge, cyclophosphamide per the Euro-Lupus protocol was initiated. As a result, the patient had considerable improvement in pain and was safely discharged home.

On follow-up interview with the patient after completing her cyclophosphamide treatments, she reported significant improvement in her functioning. She had advances in her ankle plantarflexion and toe flexion strength and was now able to walk again. The patient had also successfully completed a course of physical therapy which improved her mobility further. Additionally, her right foot pain was well controlled. The patient continues to follow-up closely with her providers for ongoing immunosuppressive treatment and pain management.

## 3. Discussion

Electrodiagnostic testing was crucial in our patient case which revealed a right tibial mononeuropathy and did so with diagnostic precision greater than that possible using physical examination or MRI. The electrodiagnostic findings revealed the source of the patient's pain and dysfunction while reducing likelihood of the pain being related to complex regional pain syndrome. The profound denervation seen in the first dorsal interosseous pedis correlated with the muscular edema seen on the MRI and the patient's severe foot pain. The patient's pattern of weakness and sensory loss was explained by the tibial mononeuropathy. As a result of the electrodiagnostic findings, the patient underwent sural nerve biopsy which confirmed the presence of vasculitis allowing definitive therapy to be initiated. One limitation of the data was that the contralateral soleus was not evaluated on needle electromyography. Since the patient also had an underlying generalized length-dependent sensorimotor polyneuropathy, a comparison between the two soleus muscles would have better characterized the extent of neurogenic findings that were due to the tibial mononeuropathy alone as opposed to the length-dependent polyneuropathy.

Additionally, the use of ultrasound in this patient case would have been beneficial for evaluating the tibial nerve. With advances in technology, ultrasound evaluation of peripheral nerves leads to both higher resolution images and increased rates of detecting pathology as compared with MRI. An additional advantage of ultrasound is that the whole length of the nerve can be evaluated cost effectively to identify and localize any neuropathology [3]. In our patient case, the tibial mononeuropathy localized just proximal to the tibial take-off branches to the soleus which was not captured on MRI limited to the foot. Furthermore, specific morphologic changes seen on ultrasound in injured nerves can provide insight into the etiology. In vasculitic neuropathies, ultrasound evaluation of affected nerves shows enlarged cross-sectional areas which may prompt additional workup into vasculitis [4]. Ultrasonographic Tinel sign can also be used to reproduce the patient's symptoms and guide next steps in management. In a case report involving a patient with mononeuritis multiplex affecting the radial nerve, the authors identified a segment of the nerve on ultrasound which had an abrupt increase in the cross-sectional area. Ultrasonographic Tinel sign over this area consistently reproduced the patient's reported dysesthesias, and radial nerve biopsy at that location confirmed vasculitis. The radial nerve was biopsied instead of the sural nerve because the patient had advanced disease in the lower extremities which may have yielded an inadequate pathologic specimen [5].

In contrast to peroneal mononeuropathies, tibial mononeuropathies due to mechanical factors or compression are rare. The nerve courses through the popliteal fossa in tandem with the posterior tibial artery near the center of the limb, surrounded and protected by soft tissue. After exiting the popliteal fossa, the tibial nerve runs posterior to the tibia before entering the tarsal tunnel [1]. Compared to the common peroneal nerve which runs superficially across the fibular head, the tibial nerve is well protected from external trauma. Known entities causing tibial nerve compression include posterior compartment syndrome, soleal sling syndrome, and tarsal tunnel syndrome.

Posterior compartment syndrome causes tibial nerve compression with coincident ischemia, which again highlights the shared course of the tibial nerve and artery. Most commonly, compartment syndrome is associated with severe trauma and tibial fracture. However, soft tissue injuries without fracture account for nearly a quarter of compartment syndrome. Acute exertional compartment syndrome has also been reported and typically associated with anabolic steroid use and rapid increases in athletic training [6]. Our patient's history of progressively worsening right lower extremity weakness and pain in the absence of risk factors for compartment syndrome made this clinical entity unlikely. Furthermore, a tibial mononeuropathy due to posterior compartment syndrome would cause denervation of the soleus and gastrocnemius muscles as both reside in the posterior compartment and share the same innervation. In our case, there was relative sparing of the gastrocnemius, whereas the soleus was markedly impacted.

Soleal sling syndrome leads to proximal tibial nerve entrapment; the origin of the soleus muscle forms a tendinous arch, compressing the tibial nerve as it enters the deep posterior compartment. Cadaveric and surgical studies show that, on average, the site of tibial nerve compression deep to the soleal sling is about 9 cm distal to the medial tibial plateau [7, 8]. Patients typically present with plantar numbness and loss of toe flexion with symptoms aggravated by ankle and foot plantarflexion which constricts the musculofascial arch over the tibial nerve. On examination, patients commonly report pain with gentle palpation of the posterior calf at the level of the soleal sling, a finding critical for diagnosis. In a series of patients with clinically diagnosed soleal sling syndrome, none had electrodiagnostic findings consistent with a tibial mononeuropathy suggesting that electrodiagnostic testing may not be beneficial in confirming this condition [7]. In contrast, our patient's electrodiagnostic findings confirmed a severe tibial mononeuropathy making this diagnosis unlikely.

Tarsal tunnel syndrome involves distal compression of the tibial nerve as it runs deep to the flexor retinaculum at the postero-inferior medial malleolus. Patients may present with paresthesias, dysesthesias, and hyperesthesias radiating from the retro-malleolar region as well as weakness in the intrinsic flexors of the involved foot. Dorsiflexion with eversion of the ankle or Tinel's sign on the tibial nerve at the tarsal tunnel may reproduce symptoms. Etiologies for tarsal tunnel syndrome include trauma, inflammatory arthropathies, space-occupying lesions, soft tissue irregularities, or biomechanical factors [9]. In our patient, MRI of the affected foot did not reveal any structural abnormalities involving the tarsal tunnel. Furthermore, our patient had significant denervation of the soleus muscle which further excluded a pure tarsal tunnel syndrome.

Since our patient's tibial mononeuropathy did not correspond to known tibial nerve compression syndromes, noncompressive etiologies of tibial nerve injury such as vasculitis were strongly considered. Systemic vasculitides are a group of diseases that involve the inflammation of blood vessels. Peripheral neuropathies such as mononeuropathy multiplex (single or multiple) or distal symmetric polyneuropathy can manifest at the onset of illness and be present in over half of small-vessel vasculitis cases [10, 11]. Moreover, neuropathy can be one of the first clinical features of systemic vasculitis. In one study, 68% of patients presenting with small-vessel vasculitis-related mononeuropathy multiplex and distal symmetric polyneuropathy reported neuropathy pain [12]. These findings correlated well with our patient case. Our patient was at an increased risk of developing a systemic vasculitis considering her history of immune dysregulation. She presented with neuropathy pain described as “crushing and burning,” and sural nerve biopsy later confirmed the presence of small-vessel vasculitis. Furthermore, our patient was found to have both length-dependent sensorimotor polyneuropathy along with a superimposed tibial mononeuropathy, fitting the known association between these conditions and small-vessel vasculitis.

The pathophysiology of vasculitic neuropathy involves inflammation and subsequent ischemia of vasa nervosum which leads to axonal damage. The larger motor and sensory nerves are affected more often because they are more vulnerable to ischemic damage [11]. Ischemic nerve lesions due to a vasculitic neuropathy lead to unequal fiber loss between and within nerve fascicles with multiple lesions of different ages spread along the nerves [13]. In a study among a small-vessel vasculitis patient population, the peroneal nerve was most commonly involved followed by tibial, median, ulnar, radial, and sciatic nerves. All patients' electrophysiologic studies revealed axonal damage [12]. In another study focusing on granulomatosis with polyangiitis (a small-vessel vasculitis), the peroneal nerve was again noted to be the most commonly involved one followed by the tibial nerve [2]. These findings reflected our patient case well. Our electrodiagnostic study localized the tibial mononeuropathy to a nonentrapment site, which is consistent with the patchy and fascicular pathophysiology in vasculitic neuropathies. It is important to consider vasculitic tibial mononeuropathies on the differential diagnoses considering that they are second in frequency only to vasculitic peroneal mononeuropathies.

In conclusion, this case illustrates several important points. Compartment syndrome, soleal sling syndrome, and tarsal tunnel syndrome are entities known to cause compressive tibial mononeuropathies. However, these are rare due to the deep, protected anatomic course of the tibial nerve in the lower extremity. When a tibial mononeuropathy is in question, particularly if severe, it is important to thoroughly evaluate for noncompressive etiologies. Systemic vasculitides, particularly those affecting the small vessels, are known to cause mononeuropathies or multiple mononeuropathies. The tibial nerve is commonly affected in small-vessel vasculitis and is second in frequency only to the peroneal nerve. It is important to maintain a high index of suspicion for vasculitic neuropathies as these may be the patient's presenting signs and symptoms. Due to the patchy and fascicular pathophysiology of vasculitic neuropathies, unsurprisingly, these may not localize to more well-defined entrapment sites on electrodiagnostic testing. Electrodiagnosis serves a critical role in evaluating vasculitic neuropathies to better localize the lesion as well as investigate competing differential diagnoses. Ultrasound evaluation for peripheral nerve pathology is also a powerful tool by providing a cost-effective way to evaluate nerves along their entire anatomic course and insight into the underlying cause of injury. By combining a solid foundation in anatomy, pathophysiology, and electrodiagnosis with clinical acumen, electromyographers are able to maximize their impact on patient care.

## Figures and Tables

**Table 1:** Electrodiagnostic data.

Nerve conduction	Amplitude (*µ*V or mV)		Latency (ms)		Conduction velocity (m/s)			
	Right	Left	Right	Left	Right		Left	
Sural sensory								
Stimulate calf, record ankle (14 cm distance)	6.0 (6–47)	7.2 (6–47)	3.4 (3.2–4.2)	3.4 (3.2–4.2)	48.3 (>40)		53.8 (>40)	
Medial plantar sensory								
Stimulate foot, record ankle	No response	No response	—	—	—		—	
Superficial peroneal sensory								
Stimulate ankle, record foot (10 cm distance)	5.6	3.8	2.8	3.1	—		—	
Peroneal motor								
Stimulate ankle, record EDB (9 cm distance)	3.8 (2–12)	3.3 (2–12)	3.8 (3.3–6.1)	4.1 (3.3–6.1)	—		—	
Stimulate below knee, record EDB	3.7	3.1	8.6	9.0	51.0 (41–56)		48.0 (41–56)	
Stimulate above knee, record EDB	3.6	3.9	10.5	11.0	52.6		50.0	
Tibial motor								
Stimulate ankle, record AH (8 cm distance)	0.3 (3–26)	1.8 (3–26)	6.0 (2.7–6.1)	4.4 (2.7–6.1)	—		—	
Temperatures: right calf: 34.8°C, left calf: 33.0°C								

Needle electromyography								
Spontaneous activity					Voluntary activity				
Muscle (right)	Insertional activity	Positive sharp waves and fibrillations	Fasciculations	Amplitude	Duration	Polyphasia		Recruitment
FDIP	Increased	Sustained 3+	0	—	—	—		0
Anterior tibialis	Slightly increased	Unsustained 0	0	Normal	Increased 2+	Increased 2+		Decreased 3+
Medial gastrocnemius	Normal	0	0	Normal	Normal	Normal		Decreased 2+
Soleus	Increased	Sustained 2+	0	Normal	Increased 2+	Increased 2+		Decreased 3+
Short head of biceps	Normal	0	0	Normal	Normal	Normal		Normal
Gluteus medius	Normal	0	0	Normal	Normal	Normal		Normal

Nerve conduction studies: sensory study values are recorded in *µ*V, and motor study values are recorded in mV. Note the markedly decreased amplitude of the right tibial motor response as compared with the left. Reference lab values are indictaed in parentheses. EDB = extensor digitorum brevis; AH = abductor hallucis. Needle electromyography: note the marked denervation of the soleus with relative sparing of the medial gastrocnemius and normal short head of the biceps. All voluntary activity was performed by the patient with normal effort. FDIP = first dorsal interosseous pedis.

## Data Availability

The numerical data used to support the findings of this case report are included within the article and the corresponding citations. Electrodiagnostic data used to support the findings of this case report were collected by the authors, and requests for this data should be made to the corresponding author.
